# Causal biological network database: a comprehensive platform of causal biological network models focused on the pulmonary and vascular systems

**DOI:** 10.1093/database/bav030

**Published:** 2015-04-17

**Authors:** Stéphanie Boué, Marja Talikka, Jurjen Willem Westra, William Hayes, Anselmo Di Fabio, Jennifer Park, Walter K. Schlage, Alain Sewer, Brett Fields, Sam Ansari, Florian Martin, Emilija Veljkovic, Renee Kenney, Manuel C. Peitsch, Julia Hoeng

**Affiliations:** ^1^Philip Morris International R&D, Philip Morris Products S.A. Quai Jeanrenaud 5, 2000 Neuchâtel, Switzerland, ^2^Selventa, One Alewife Center, Cambridge, MA 02140, USA and ^3^Applied Dynamic Solutions, LLC, 220 Davidson Avenue, Suite 100, Somerset, NJ 08873, USA

## Abstract

With the wealth of publications and data available, powerful and transparent computational approaches are required to represent measured data and scientific knowledge in a computable and searchable format. We developed a set of biological network models, scripted in the Biological Expression Language, that reflect causal signaling pathways across a wide range of biological processes, including cell fate, cell stress, cell proliferation, inflammation, tissue repair and angiogenesis in the pulmonary and cardiovascular context. This comprehensive collection of networks is now freely available to the scientific community in a centralized web-based repository, the Causal Biological Network database, which is composed of over 120 manually curated and well annotated biological network models and can be accessed at http://causalbionet.com. The website accesses a MongoDB, which stores all versions of the networks as JSON objects and allows users to search for genes, proteins, biological processes, small molecules and keywords in the network descriptions to retrieve biological networks of interest. The content of the networks can be visualized and browsed. Nodes and edges can be filtered and all supporting evidence for the edges can be browsed and is linked to the original articles in PubMed. Moreover, networks may be downloaded for further visualization and evaluation.

**Database URL:** http://causalbionet.com

## Introduction

A paradigm shift in data generation and collection, and the growing number of scientific journals published have contributed to the exponential rate of growth in peer-reviewed publications. For example, in 2013, MEDLINE counted over 20 million citations from 5640 indexed journals out of more than 20 000 journals that existed at that time (1). In addition, modern profiling technologies can measure subtle changes in tens of thousands of molecular species, be it in DNA and RNA sequences and their quantities, proteins, lipids, metabolites and epigenetic marks among others. All these data can be stored in databases. This wellspring of public data and knowledge has led to new challenges, such as keeping track of new knowledge and being able to obtain an overall picture of the knowledge accumulated in a particular field. Computational approaches, including *in silico* models, databases and standardized languages that leverage prior knowledge derived from the literature and process content-rich biological data sets have been used to create a framework to address these challenges. Pathways and network-based representations of biological mechanisms are flourishing and can capture knowledge in disparate ways. Some of these databases are summarized in Table 1. The presence and definition of boundary conditions, types of representation, languages used and depth of context annotation vary widely across repositories.

**Table 1.** Databases that provide pathways and network-based representations of biological mechanisms

	CBN	KEGG	Reactome	BioCarta	Wiki-pathways	SPIKE	UCSD signaling gateway	NCI pathway interaction database	NetPath
Species [human (Hs); mouse (Mm); rat (Rn)]	Hs, Mm*, Rn*	>20 species	Hs (curated)	Hs, Mm	>25 species	Hs	Hs, Mm	Hs	Hs
			+20 species (inferred)						
Literature support displayed									
At edge level	✓					✓	✓	✓	✓
At pathway level		✓	✓	✓	✓			✓	
Defined biological boundaries									
Species	✓	✓		✓	✓	✓		✓	✓
Tissue	✓								
Disease context	✓	✓			✓				✓
Biological pathways	✓	✓	✓	✓	✓	✓		✓	✓
Manual curation	✓	✓	✓	✓	✓	✓	✓	✓	✓
Data-driven enhancement	✓						✓		✓
Crowd curation	✓			✓	✓				±
Directional edges	✓	✓	✓	✓	✓	✓	✓	✓	✓
Multiple types of entities	✓	✓	✓	✓	✓	✓	✓	✓	
Interactive visualization	✓		✓		✓				
Computable	✓	✓	✓						
Available for download	✓	✓	✓		✓	✓	✓	✓	✓
Size	>120 network models	>450 pathway maps	>1400 pathways (Hs)	>350 pathways	>430 pathways	>25 curated pathways	∼3500 proteins and their proximal connections	>135 NCI-Nature curated pathways (+Reactome + BioCarta)	>30 curated pathways (immune signaling/cancer)

*coming soon

Among the most used databases of pathways/networks, the Kyoto Encyclopedia of Genes and Genomes (KEGG) was one of the first databases of biological signaling pathways made freely available to the general scientific community (2, 3). First published in 1995 to describe metabolic pathways, recent additions to the KEGG collection include general signal transduction and disease pathways. The November 2014 version of KEGG Pathways contained 467 signaling modules, organized into seven high-level domains. Each pathway map provides a set of published articles that serve as the conceptual reference framework for the over-arching biology described in each pathway. The University of California, San Diego (UCSD) Signaling Gateway is another resource for biological knowledge centered on individual molecules and their associated local signaling network. Each of the >8000 molecule pages described by the UCSD Signaling Gateway (http://www.signaling-gateway.org/molecule/) provides details on molecules with proximal connections within signaling cascades driven by the reference molecule. Content is derived from both domain experts and curated from highly structured data sets (e.g. protein interactions) using automatic annotations from publicly available data sources (e.g. UniProt and GenBank) (4, 5). Annotations describe ‘states’ (e.g. complexes, phosphorylation state, physical location) and ‘transitions’ (relationships between initial state and final state), both of which are referenced by published literature. The Signaling Pathway Integrated Knowledge Engine (SPIKE) (http://www.cs.tau.ac.il/∼spike) is a database of curated human signaling pathways associated with an interactive software tool that facilitates customization of the layout. The SPIKE database describes 28 pathways organized into seven biological areas (Map Topics). The graphical pathway maps are laid out by domain experts to describe relationships backed by published scientific findings. Notably, the SPIKE database contains relationships drawn from other large signaling databases including KEGG, Reactome (6) and several protein–protein interaction networks. In addition to the available prearranged pathways, users can create and export their own networks from the content available in the SPIKE database.

**Figure 1.**
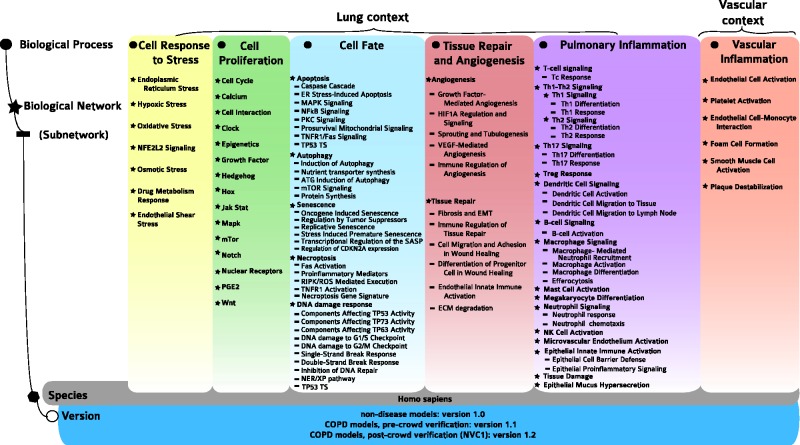
Network models and constituent subnetworks in the CBN database. For each of the six biological processes (marked by circles), the biological networks (marked by stars), and, whenever relevant, subnetworks (marked by lines) describing distinct signaling pathways are listed. Currently, a total of 123 network models with 1–3 different versions each are deposited in the CBN database. Note that even though the endothelial shear stress network was built with the other stress models, its context is exclusively vascular.

Here, we report the causal biological network (CBN) database, a collection of modular, evolving, manually curated, biological expression language (BEL)-scripted causal network models that describe signaling pathways relevant in diseased and nondiseased pulmonary and vascular tissues. Structurally, the CBN collection is organized into the following biological areas (Figure 1): cell response to stress, cell proliferation, cell fate (including DNA damage response, autophagy, senescence, apoptosis and necroptosis), tissue repair and angiogenesis, pulmonary inflammatory processes, vascular inflammatory processes and chronic obstructive pulmonary disease (COPD)-specific networks. Details of these network models have been published previously (7–12).

## CBN models are scripted in the biological expression language

The modeling approach used to build the CBN models (described below) uses BEL. The basic concept of BEL is the conversion of prior knowledge derived from either the literature or content-rich biological data sets to a common biological language to create a framework for the generation of new hypotheses. BEL was designed to capture biological cause-and-effect relationships with associated experimental context from disparate sources, and to allow the encoding of directional relationships within computable biological network models. One of the most important features of BEL is that it is both human readable and computable. Another important feature is that rich contextual information, including species, tissue, experiment type, may accompany specific statements. This is particularly important to model for instance diseases or individual responses to drugs that may be associated with specific phenotypes or genotypes (13). There are two ways to integrate BEL-scripted biological knowledge into a useful structure, depending on the needs of specific applications. One application is the creation of structured biological signaling pathways. Another application is to guide data analysis and generate novel predictions by applying statistical algorithms that incorporate curated relationships into a computable framework using, e.g. reverse causal reasoning (RCR) (14). OpenBEL, an open consortium for the BEL framework, provides tools and resources to script and edit using BEL (BEL editor), to visualize BEL-scripted network models [Knowledge Assembly Model (KAM) navigator] and for the computable analysis of network models (Whistle). All these tools and resources are freely available on the OpenBEL portal (http://www.openbel.org).

Other open representation standards such as Biopathways Exchange Language (BioPAX) and Systems Biology Markup Language (SBML), while in broader use, may be less well suited than BEL for the purpose of modeling causal networks which CBN models aim at. BioPAX (15) was designed to facilitate exchange of pathway information and is amenable to representing relationships from the scientific literature. Although BioPAX models are more precise at the biochemical level of description than the discrete relationships represented by BEL (16), they are less flexible in annotation of relationships with contextual information. SBML (17) was designed for quantitative computational models. While qualitative models can now be built with the qual package (18), SBML was not designed for capture of information from the scientific literature nor for integration of mechanism fragments from various sources. Another key feature of BEL compared to both BioPAX and SBML is human readability. The integration of mechanism fragments extracted by natural language processing into pathways can be automated to some extent (19), but is likely to require some human evaluation and intervention, which is facilitated by the readability of the BEL.

**Figure 2.**
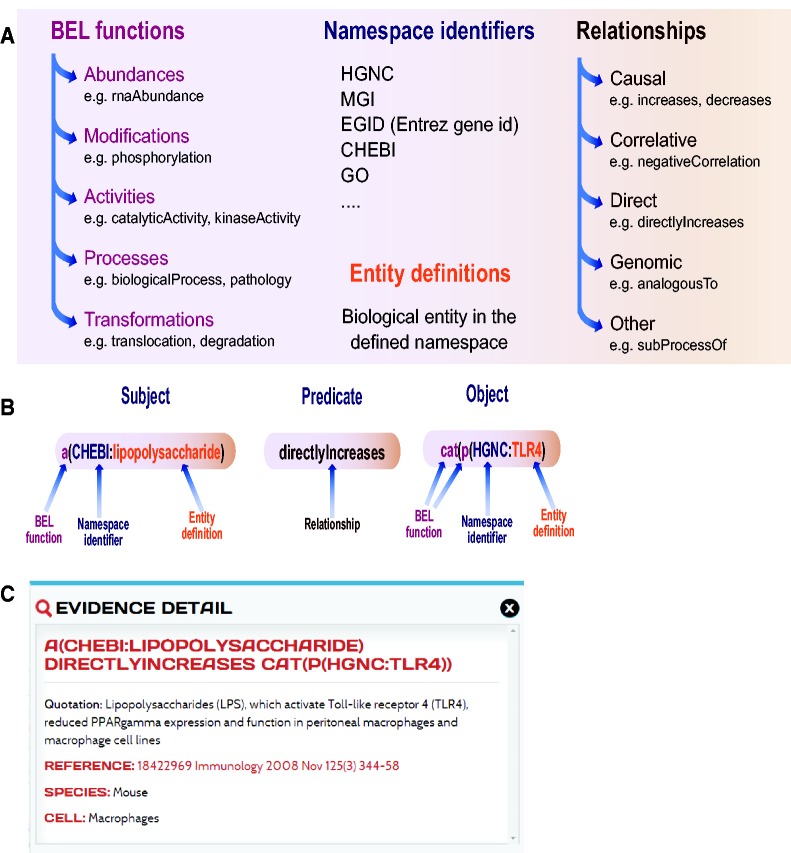
Biological Expression Language (BEL). (**A**) Elements of BEL: BEL functions, namespace identifiers, and entity definitions compose each node name that can be either subject or object in the relationship. (**B**) Example of a BEL semantic triplet composed of a subject (abundance of LPS), a relationship or predicate (directly increases) and an object (catalytic function of TLR4). (**C**) Detailed information on one of the underlying pieces of evidences and a link to the original article and context in which the relationship was demonstrated in the article.

Knowledge is expressed as BEL statements and subsequently stored in BEL documents. BEL statements are defined as semantic triplets: subject–predicate–object, where the subject and object are BEL terms and the predicate is one of the BEL relationship types (e.g. increases, decreases) that can also be represented by a symbol (Figure 2A). Currently, several types of biological entities are represented as BEL terms, which can include abundances (e.g. abundance of protein, RNA, complexes), modifications (e.g. phosphorylation) and activities (e.g. transcriptional activity, kinase activity), and processes (e.g. gene ontology biological process, phenotype). The BEL term for a protein is usually composed of the protein name acronym and a namespace identifier that indicates the database or ontology where the acronym was defined. In addition to the precise description of the terms and relationship, each BEL statement can be annotated to express knowledge about context of the statement such as information about the biological system, experimental method and/or appropriate literature citation. An example of a BEL statement is shown in Figure 2B. In this example, the statement ‘a(CHEBI:lipopolysaccharide) directlyIncreases cat(p(HGNC:TLR4)’ encodes information about the subject, namely the abundance of the lipopolysaccharide (LPS), which is identified by its namespace identifier from the Chemical Entities of Biological Interest (ChEBI) database (20). The relationship (the predicate of the triplet) scripted in this example is a direct increase, implying that LPS directly interacts with TLR4 to increase its activity. The object for this example is the catalytic activity of the protein identified in the HGNC namespace by its abbreviation toll-like receptor 4 (TLR4). The BEL document encoding this statement was annotated with eight pieces of evidence text from peer-reviewed articles that are maintained with the BEL statement and are viewable on the web-based platform.

## Unique properties of CBN models

While existing resources described in the preceding section and in Table 1 share similar advantages in terms of references to prior knowledge and exportability, the CBN models offer several distinct advantages that stem from the strategy followed for their construction (summarized later):

- The network models were built within defined biological and context boundaries: signaling pathways and molecules important to define biological processes of interest are scoped out based on experts’ knowledge and review articles. Moreover, context boundaries (species, tissues, cell types, experiment types, disease) are set for the provenance of the pieces of evidence and datasets used for network building. This is unlike other common approaches for building pathway or connectivity maps where connections are often represented without regard for tissue or disease contexts.
- In addition to the pathways evident in the scientific literature, CBN models also include data-derived nodes identified through RCR analysis of datasets of interest using the Selventa Knowledgebase (http://selventa.com/), which contains over two million curated relationships (for more details, see the network construction section below).
- CBN models provide a degree of precision on the biological entities that is not found in comparable resources. Because of BELs’ representational flexibility, the networks can capture a wide range of biological molecules including proteins, DNA variants, coding and non-coding RNAs, chemicals, lipids and methylation states or other modifications (e.g. phosphorylation). Moreover, this allows multiple (omics) data types to be mapped concurrently to the network, which aids the biological interpretation of complex data sets. In addition to concrete molecular entities (e.g. proteins, mRNAs), more complex biological processes (e.g. macrophage activation, apoptosis) are causally represented in the networks.
- The nodes in the network are connected by causally related edges. This feature not only allows the biological intent of the network model to be easily digested by a human scientist, but also enables inference and computation on the network as a whole. For example, the freely available tool Whistle allows for the computational identification of potential upstream controllers for a set of differentially expressed genes along with a set of statistical measures relating to the confidence of the prediction. These upstream controllers can then be mapped onto the CBN models to identify biological pathways that are activated/repressed in a given experiment.
- Most causal edges in the network models are supported by at least one (often multiple) literature reference. In addition to these citations, edges have been annotated with a variety of metadata, including species, and tissue and cell type, and further context-specific information can be added. Because the edges (cause and effect relationships) in the network model are supported by published scientific findings, each network model is anchored to the scientific literature for the biological process being modelled.
- The networks are evolving (see below) and can be modified to represent specific species and/or tissue types by the application of context-specific filters and selective addition of new context-specific evidence that can be updated as new knowledge becomes available.
- The network models can be browsed on the CBN database website and downloaded in a portable network visualization format to allow network visualization using Cytoscape (21) and other tools that are freely available.

## Construction of the first version of the CBN models

**Figure 3.**
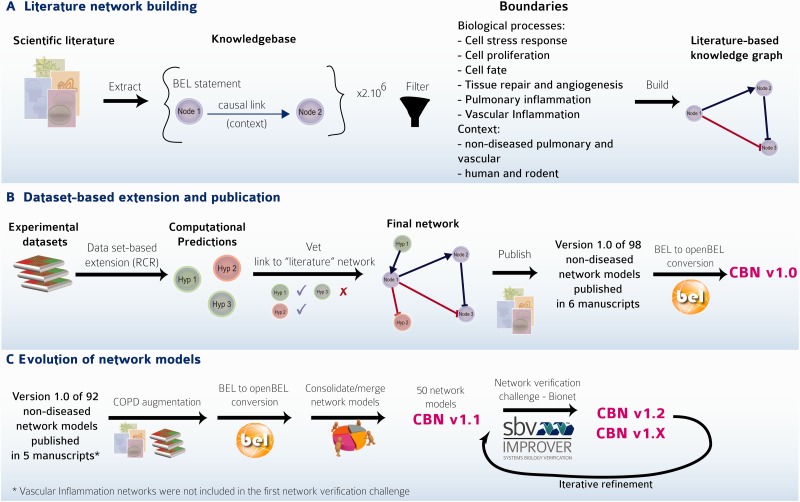
The CBN models. (**A**) The CBN models are made up of causally connected BEL relationships extracted from selected evidence text in the scientific literature. (**B**) Models are enriched with hypotheses generated by reverse causal reasoning (RCR) on data sets of interest. (**C**) Models can evolve and be augmented to include, e.g. COPD-relevant mechanisms. Networks were and continue to be subjected to crowd verification under the umbrella of the sbv IMPROVER network verification challenge. Multiple versions of the networks have been deposited into the CBN database to keep the history and to reflect the iterative refinement of the networks.

The first versions of 98 network models (Version 1.0) were constructed and evaluated following the strategy reported previously (7–12). This strategy (Figure 3A and B) is summarized briefly here:

1. Careful selection of model boundaries, i.e. selection of appropriate tissue/cell context (respiratory tract tissues and vascular tissues in a nondiseased context) and biological processes to be included in the model.
2. Review of scientific literature to extract relevant causal relationships, resulting in the construction of the literature model scaffold composed of nodes and edges, representing causal relationships between the nodes extracted from the Selventa Knowledgebase.
3. In the network verification and extension step, RCR was used to mine molecular profiling data in the context of the Selventa Knowledgebase (14). RCR is a reverse engineering algorithm to identify biological mechanisms that are statistically significant explanations for differential measurements in a molecular profiling data set. RCR analysis of relevant (i.e. within defined boundaries) data sets allowed the identification and inclusion, where appropriate, of additional biological processes derived from these experimental data that may not be apparent from evaluation of scientific literature dedicated to the biological process of interest. Attentive expert curation during the integration of each newly identified mechanistic node into evolving networks that are iteratively derived from data sets ensured the continuity of relevant boundary conditions during network construction.

The final network models reflect the value of the combined use of molecular profiling data with careful considered use of prior biological knowledge of cause-and-effect relationships

## Evolution of CBN models using an innovative crowd-sourcing verification platform

The network models were built to represent an extensive body of published literature and datasets. As knowledge grows, the network models need to evolve to better represent and integrate relevant existing knowledge. To verify and enhance previously built biological network models, the sbv IMPROVER initiative, which was set up to verify methods and data used in systems biology (22), developed the Network Verification Challenge (NVC) using a social networking approach to generate high-quality curation results (23, 24). This crowd-verification process was designed to assemble the knowledge of domain experts and focus the critical minds of biologists from across multiple fields of biology to effectively and efficiently review the evidence available in the literature to improve biological network annotations. Details of the modifications made to the biological networks prior, during, and after the NVC are detailed in a dedicated article (24) and briefly summarized here.

**Figure 4.**
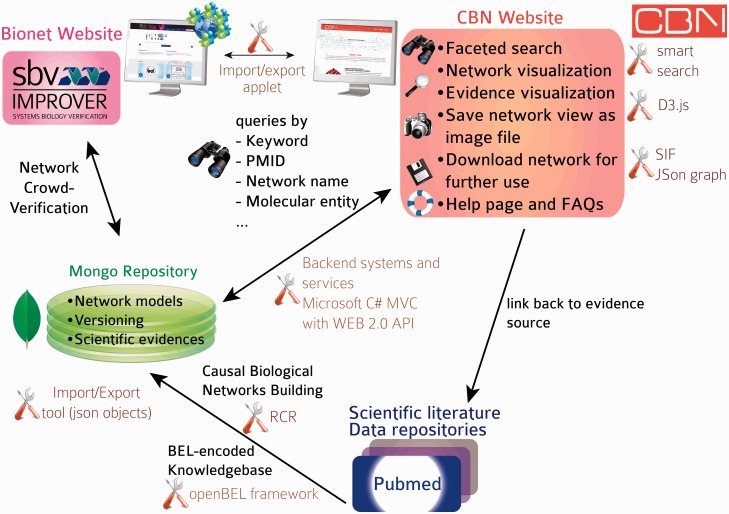
Database and website architecture. Knowledge is extracted from the scientific literature, scripted in BEL and stored in a knowledgebase. All networks are stored as JSON objects in a MongoDB repository with rich metadata (network title, description, version). Networks can be subjected to crowd verification in sbv IMPROVER and are accessible from the CBN database website. A smart search allows users to find relevant network models by searching, e.g. for keywords, molecular entities, biological processes in the nodes list, network title and network description. Networks returned in a search may be exported in different file formats or displayed in a network viewer powered by d3.js (a JavaScript library for manipulating documents based on data) from which additional functionalities are available, such as exporting specific network views as images. All underlying pieces of evidence can be browsed and are linked to the original scientific literature.

The initial focus of the NVC revolved around mechanisms implicated in COPD disease pathophysiology. Therefore, before the start of the challenge, COPD-relevant mechanisms, including B-cell and T-cell activation, airway remodeling, extracellular matrix (ECM) degradation, efferocytosis, mucus hypersecretion and emphysema were added to the published ‘non-disease’ pulmonary networks. In addition, prior to deploying the COPD-enhanced biological networks on the NVC website on Bionet (http://bionet.sbvimprover.com) for verification by the scientific community, the set of ‘pulmonary’ networks were agglomerated to yield a more concise set of 50 networks that combined related/complementary cellular pathways. This set of 50 network models is also available in the CBN database as version 1.1 of the network models. The relationships between versions 1.0 and 1.1 are described in a network hierarchy figure (Figure 1) and in the Supplementary data describing all available network models of the CBN database (Supplementary Material). Improvements made to the network models during the NVC-included submission of new pieces of evidence, additional literature publications to support existing network edges, as well as submission of new biological edges with supporting evidence for relationships that were not represented in the original networks. When networks are refined, they are imported into the CBN database as new versions (Figure 3C). Thus, conceptually, Bionet and the CBN platform are linked intrinsically and were developed in parallel so that the CBN database constitutes a repository of all versions of the network models and Bionet contains a single version of the network that is open for crowd verification (Figure 4).

## CBN database structure and statistics

The architecture and tools supporting the CBN database are summarized in Figure 4. Briefly, data were curated from the scientific literature and databases and scripted in BEL to constitute the Selventa Knowledgebase on which the networks were built. RCR allowed more comprehensive networks to be built, including nodes that may not be easily retrieved from scientific literature searches based on a specific biological mechanism. Network models are stored as JavaScript Object Notation (JSON) graph objects, a new format based on JSON objects that was developed to capture basic graph structures in a convenient to use format (http://json-graph-format.info/). In addition to the information on nodes and edges, this format allows the use of rich metadata, including (but not restricted to) graph layout, style, and annotation of edges. Both the network models and the supporting evidence are imported and stored in a MongoDB repository (www.mongodb.org). MongoDB was chosen because of its native JSON storage and greater flexibility in data schemas. Providing a native JSON store works indeed very well for application stores that are web or mobile-based. Relational databases are much more rigid in their schemas and any change to the data schemas require a great deal of refactoring and data migration. The final factor in the selection process was the limited need for intertable/database relationships. Most of the data required for an application operation is totally encapsulated by one ‘document’ or record in the MongoDB. It does not require flexible joins between normalized datasets which is where relational databases like Mysql provide significant value. An applet allows the CBN database to communicate (import/export networks) with the Bionet platform. Backend systems and services (detailed in the next section) allow for a very responsive website that can display data from the CBN database. An icon was specifically designed for each network to represent in a graphical way the biological mechanism that is modelled in the network. The full content of the first release of the CBN database is summarized in the Supplementary data, including network name, biological process, network description, version and network icon. Statistics of the content of the first CBN database release are summarized in Table 2. The database will be updated bi-monthly to include up-to-date underlying pieces of evidence, new network versions, and possibly new network models.

**Table 2.** Statistics of the CBN database (August 2014 release)

	Total number	Number of unique instances^a^
Network models	123	123
Nodes	14 755	3739
• mRNA abundance	743	465
• Protein abundance	7464	1583
• Molecule abundance	736	195
• Complex abundance	696	227
• Kinase activity	1445	180
• Catalytic activity	1376	365
• Transcriptional activity	924	166
• Biological process (GO)	531	151
• Pathology	115	41
• Secretion	145	92
• Degradation	99	53
• Others	481	221
Edges	21 076	10 116
• Causal	13 702	7290
○ increases	8010	4640
○ directlyIncreases	3227	1340
○ decreases	1650	965
○ directlyDecreases	815	345
• Noncausal	7374	2826
Evidence	535 192	80 900
• Human	391 236	60 482
• Mouse	100 286	14 462
• Rat	42 330	5429
• Species not annotated	1280	527

^a^Differences between the total number and the number of unique network instances reflect the presence in the CBN database of different versions of a number of networks that have the same name.

## Functionalities available in the CBN web-based platform

**Figure 5.**
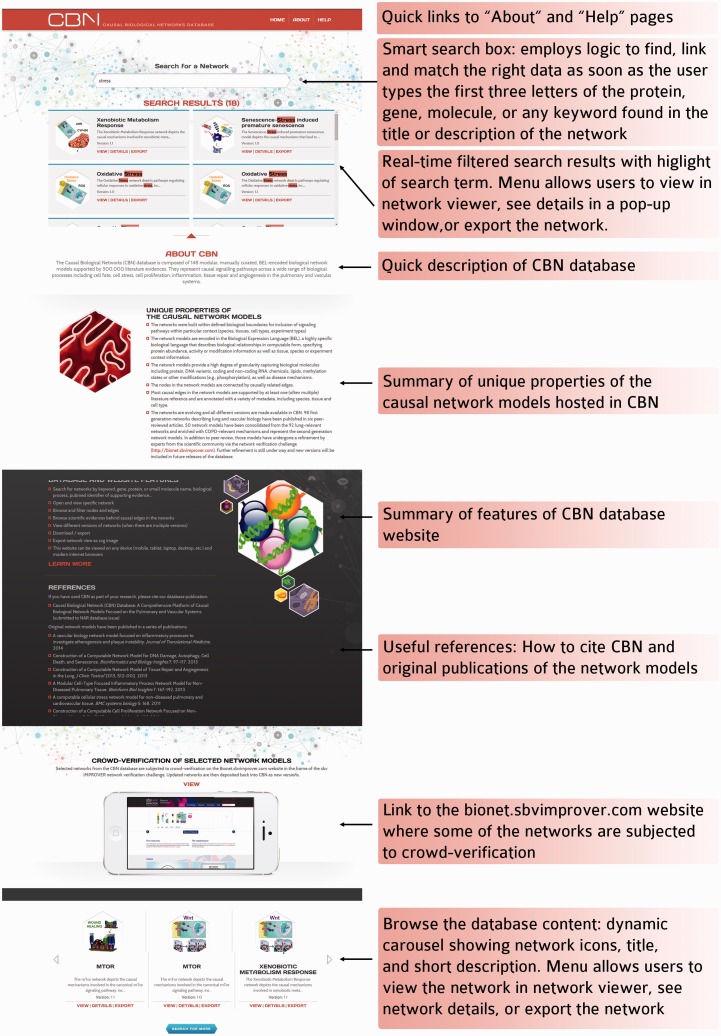
Functional and ease of access to information on the CBN database website homepage.

**Figure 6.**
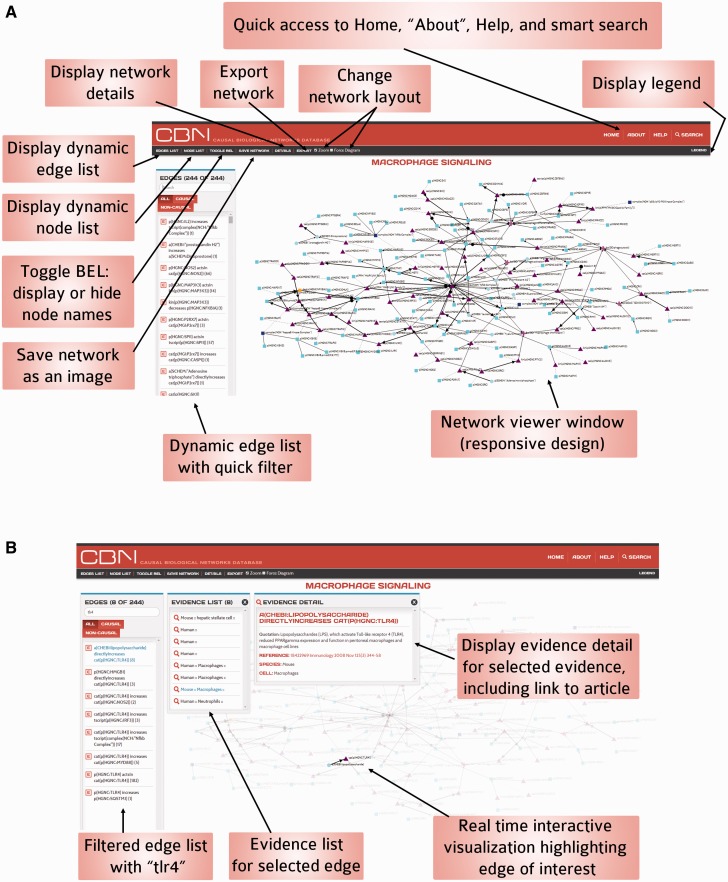
Functionality of the interactive network visualization platform in the CBN database. (**A**) For less-crowded visualization, it is possible to hide node names using the Toggle BEL feature. (**B**) Users can select nodes and/or edges and the visualization updates instantly to highlight the selection and the rest of the network fades into the background.

The CBN website was designed to give the best possible user experience so that users could benefit from the rich resources available in the CBN database. The main features that are available on the homepage and network visualization page are summarized in Figures 5 and 6.

## Design

The homepage (Figure 5) allows for quick access to all functionalities on the website: smart search, link to Help page, summarized description of concepts and functionalities, links to Bionet and Carousel dynamic display of all networks.

Responsive web design was implemented to allow users to view the website on any device (e.g. mobile, tablet, laptop, desktop). The layout and images respond to the user’s behavior and environment based on screen size, platform, and orientation. Moreover, network icons were designed to give a quick visual overview of the biological processes that are modelled in the respective networks.

## Browse/search the database content

Because the content of the CBN database is already dense and is intended to grow further, and because the content may be partially redundant (i.e. single entities and/or relationships may be present in multiple network models and across versions), it is of utmost importance for usability that the database has efficient search capability. Therefore, flat rows of data are converted to multifaceted entities, and dynamically rendered to allow for sorting and filtering. Then a smart search employs logic to find, link and match the right data as soon as the user types the first three letters of the network, protein, gene or any keyword to generate instantly meaningful search results. In the search results window that is dynamically filtered as the user types in the search box, the network icon, network name, network version and the beginning of the network description are displayed together with a choice of actions the user may take: (i) go to the network visualizer to browse the network, (ii) display more details about the network or (iii) download the network for offline use. All queries are run directly against the most current data, which is updated bimonthly. The user may search networks by keywords; e.g. network title, molecule name, PubMed identifier, protein and genes.

## Network viewer

The network viewer, powered by d3.js, allows real-time interactive data visualization that connects the visualization to the nodes and edges list for a network model of choice (Figure 6). The relationships in the data are made visually explicit by a consistent code summarized in the legend. An initial model layout is set up (‘fixed’ layout) and the user can zoom in and out. Because some networks are big, in some instances it is useful to use another layout and to be able to move nodes as the user sees fit; in such cases, the force-directed diagram layout allows users to select and view specific nodes and edges one step out. For less crowded visualization, it is also possible to hide node names using the Toggle BEL feature (Figure 6A). Selecting a node from the node list highlights associated edge connections. Similarly, selecting an edge from the list highlights associated supporting evidence in the view and displays evidence details (including name of edge, scientific article quotation, reference article, link to the PubMed abstract, and context annotation) for a selected evidence. As users select nodes and/or edges, the visualization updates instantly to highlight the selection and the rest of the network fades away (Figure 6B). It is possible for the user to get direct access to other versions of the network by following a link on the network viewer page.

## Export content for offline use

Two features have been implemented to export content from the CBN database:

- A download/export network function that allows users to save the network in two different formats is the most flexible:
  ○A simple list of nodes and edges can be saved in Simple Interaction Format (SIF) for importation to Cytoscape, Excel or other tools.
  ○A network can be saved in JSON graph format to retain advanced features, such as layout and evidence information. This file can then be opened using a Cytoscape plugin available on the cytoscape app store. For more detail on the structure of this file format see http://json-graph-format.info/
- Any view of the network created by a user on the platform can be saved as an image in Scalable Vector Graphics (SVG) format.

## Help and FAQs

As BEL-scripted causal biological networks are not (yet) widely used within the scientific community, a help page was built to provide useful resources to learn more about the BEL language and the network models. In particular, within the framework of the sbv IMPROVER NVC, webinars and short videos that summarized all the useful concepts have been made available.

## Use and future directions

**Figure 7.**
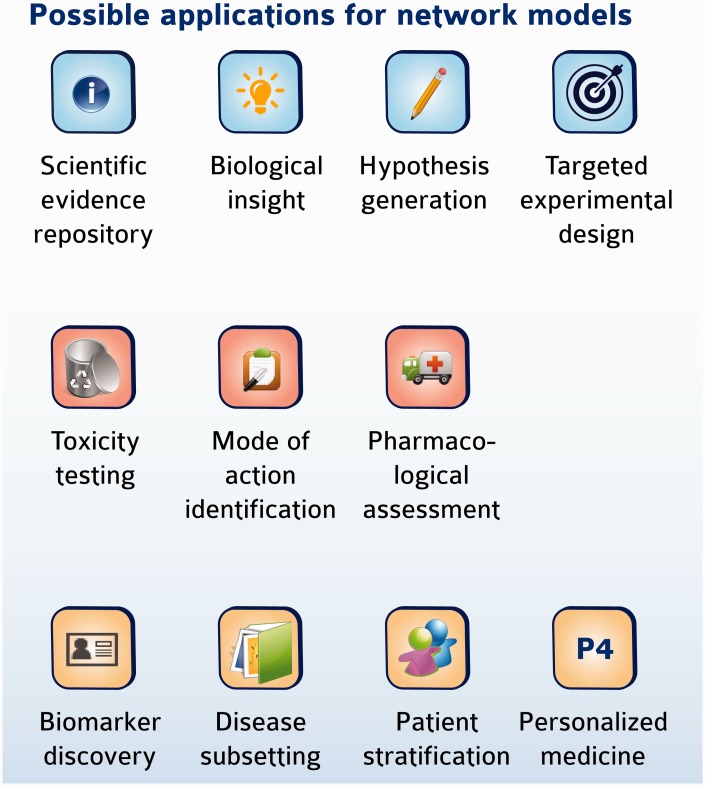
Possible application of network models. Network models may be useful in diverse applications ranging from mechanistic investigation of biology to clinical relevance and personalized medicine.

The biological information contained in the CBN models can be used in a variety of research applications, some of which are summarized in Figure 7. In a simple case, a list of differentially expressed genes could be mapped to network models to help identify perturbations in biological pathways that may be modulated following an experimental challenge, similar to the pathway enrichment approach using KEGG maps. The inherent computability conferred by encoding networks in BEL allows them to be interrogated and traversed to explore relationships and identify pathways that connect biological entities. We have described previously the development of statistical approaches to predict and interpret biological hypotheses from high-dimensional data sets such as those derived from microarray profiling studies (14). One such approach is the use of RCR to identify hypothetical causes of downstream transcriptional changes. The open tool Whistle together with a sample open-source knowledgebase are available to perform such a task from the OpenBel consortium webpage (www.openbel.org) (14). In a more complex example, we recently used the set of CBN models (∼v 1.0) with internally developed algorithms and the Selventa Knowledgebase to compute the network perturbation amplitude (NPA) following simple experimental exposures (25). In this example, we were able to detect dynamic changes in the amplitude of perturbation in a network model describing the tumor necrosis factor-nuclear factor kappa B (TNF-NFkB) signaling pathway following TNF treatment of normal human bronchial epithelial (NHBE) cells *in vitro* as measured by gene expression data. Importantly, the quantitated changes in network amplitude corresponded to direct experimental measurement of NFkB nuclear translocation following TNF treatment. In similar examples, we have used the CBN models to identify changes in the cell cycle following exposure of NHBEs to a cell cycle inhibitor (26), and to identify changes in cell proliferation, inflammation and necrosis in the rat nasal epithelium following exposure to formaldehyde (27). Of interest, specific subsets of biological networks may be applied to different experimental settings ranging from cigarette smoke exposure of rats (28), to environmental toxicants analyses *in vitro* (29), or to translational biology of xenobiotic metabolism (30). These examples illustrate how the network models contained in the CBN database (or similar network databases) can identify and even quantitate biological changes induced by diverse stimuli. Moreover, as the networks are causal, the backbone topology can be leveraged by algorithms to quantitate even more precisely the overall impact of a stimulus on a biological system (31). In addition, downloading the network models and using basic Cytoscape tools provides the ability to compute a set of topological parameters for a given network, including network connectivity measures, characteristic path lengths, and neighborhood connectivities (32), enabling the formulation of hypotheses and possible target design for experimental follow-up.

We also envision that the collection of CBN models will grow thanks to the addition of other resources and/or biological processes. In the future, CBN would no longer be limited to pulmonary and vascular biology, but would be a more general purpose database. Historically, converting prior knowledge into BEL was largely a manual process that required many curators with expertise in the related fields and their full commitment to the curation task. Technologies now exist that can use state-of-the-art algorithms to recognize various text entities and collect and assemble them into BEL statements based on their context and meaning. This could promote the semi-automated assembly of BEL-scripted knowledge databases (19, 33) that could be used for the creation of new network models and for the creation of a knowledgebase on which RCR and quantitative analyses of omics datasets could be based. Importantly, even though BEL is well adapted to script biological network models as it is both human and machine-readable and requires little adjustments of linguistic tools, it is not the only language being used to represent biological knowledge. In particular, rich-text format (RTF) and Systems Biology Markup Language (SBML) have been used extensively. Even though the specifics of each language makes them better fitted to different types of application, it is important to be able to translate knowledge between languages in a manner that conserves as much of the granularity as possible. Therefore, BEL converters are being developed and will be made available through the OpenBEL consortium, which will allow the CBN database to be populated with networks created in other languages.

The repository of CBN models has now been placed in the public domain and been promoted through the sbv IMPROVER NVC; therefore, we expect that a new suite of applications will emerge as the use of these network models evolves to fill additional niches. In particular, we plan to make available an application layer that will be built on top of the networks and allow to computationally use the network models for further applications that may include toxicity testing, biomarker discovery, target identification and mode of action (MOA) investigations, as well as patient stratification in the area of personalized medicine (Figure 7).

The CBN collection represents a significant improvement over earlier iterations of biological pathway knowledge representation, particularly in the fields of vascular and pulmonary pathobiology. We anticipate that it will be useful to many investigators engaged in analysis of high-throughput data sets derived from pulmonary tissues and provides a framework for comparing results from experimental data sets to other data sets and to the literature in an annotated environment.

## Supplementary Data

Supplementary data are available at *Database* Online.

Supplementary Data
