# Supplementary material for: Causal biological network database: a comprehensive platform of causal biological network models focused on the pulmonary and vascular systems
Source: Database (Oxford). 2015 Apr 17;2015:bav030. doi: 10.1093/database/bav030 (PMC4401337; doi:10.1093/database/bav030)
Supplement: Supplementary Data [file supp_2015_bav030_index.html]

Causal biological network database: a comprehensive platform of causal biological network models focused on the pulmonary and vascular systems — Supplementary Data 

# Causal biological network database: a comprehensive platform of causal biological network models focused on the pulmonary and vascular systems

## Supplementary Data

files

**Files in this Data Supplement:**

- Supplementary Data - xlsx file
